# A Biographical Dictionary of Cotemporary American Physicians and Surgeons

**Published:** 1880-01

**Authors:** 


A Biographical Dictionary of Cotemporary American Physicians. and Surgeons. Edited by Wm. B. Atkinson, M.D., Permanent Secretary of the Am. Med. Ass. and of the Med. Society of the State of Pennsylvania, etc., etc. Second edition, enlarged and revised. D. G. Brinton Publisher, Philadelphia. 1880.

The well-known energy and enterprise of the compilers of this work is sufficient guarantee of its efficiency. It is gotten up in good style, and its general arrangement is such as will give satisfaction.

